# GABA_B_ receptor signaling in the caudate putamen is involved in binge-like consumption during a high fat diet in mice

**DOI:** 10.1038/s41598-021-98590-9

**Published:** 2021-09-29

**Authors:** Runan Sun, Taku Tsunekawa, Tomonori Hirose, Hiroshi Yaginuma, Keigo Taki, Akira Mizoguchi, Takashi Miyata, Tomoko Kobayashi, Mariko Sugiyama, Takeshi Onoue, Hiroshi Takagi, Daisuke Hagiwara, Yoshihiro Ito, Shintaro Iwama, Hidetaka Suga, Ryoichi Banno, Bernhard Bettler, Hiroshi Arima

**Affiliations:** 1 Department of Endocrinology and Diabetes, Nagoya University Graduate School of Medicine, 65 Tsurumai-cho, Showa-ku, Nagoya, 466-8560 Japan; 2 Department of Endocrinology and Diabetes, Ichinomiya Municipal Hospital, 2-2-22, Bunkyo, Ichinomiya, 491-8558 Japan; 3 Research Center of Health, Physical Fitness and Sports, Nagoya University, Nagoya, 464-8601 Japan; 4 Department of Biomedicine, University of Basel, 4056 Basel, Switzerland

**Keywords:** Obesity, Reward, Feeding behaviour

## Abstract

Previous studies suggest that signaling by the gamma-aminobutyric acid (GABA) type B receptor (GABA_B_R) is involved in the regulation of binge eating, a disorder which might contribute to the development of obesity. Here, we show that intermittent access to a high fat diet (HFD) induced binge-like eating behavior with activation of dopamine receptor d1 (drd1)-expressing neurons in the caudate putamen (CPu) and nucleus accumbens (NAc) in wild-type (WT) mice. The activation of drd1-expressing neurons during binge-like eating was substantially increased in the CPu, but not in the NAc, in corticostriatal neuron-specific GABA_B_R-deficient knockout (KO) mice compared to WT mice. Treatment with the GABA_B_R agonist, baclofen, suppressed binge-like eating behavior in WT mice, but not in KO mice, as reported previously. Baclofen also suppressed the activation of drd1-expressing neurons in the CPu, but not in the NAc, during binge-like eating in WT mice. Thus, our data suggest that GABA_B_R signaling in CPu neurons expressing drd1 suppresses binge-like consumption during a HFD in mice.

## Introduction

Obesity, which has recently become a global epidemic, is caused when energy intake overwhelms energy expenditure. Predisposing factors for obesity include excess intake of palatable and calorie-rich food, such as a high-fat diet (HFD), as well as irregular eating, such as a binge-eating disorder^1^. Binge eating is characterized by the consumption of large amounts of food in a short duration, with a loss of control from overeating^2^. Given that the prevalence of binge eating is increasing^2^, a need exists to clarify the mechanisms causing such complex feeding behavior^3^.

Feeding is controlled by both homeostatic and reward systems^4^. The latter is mainly composed of the mesolimbic system, in which dopaminergic neurons in the ventral tegmental area (VTA) project to the nucleus accumbens (NAc) and prefrontal cortex (PFC), while dopaminergic neurons in the substantia nigra pars compacta (SNc) mainly project to the caudate putamen (CPu) in the striatum^5,6^. Recent studies have shown that dysfunction of dopaminergic neurons in the mesolimbic system is associated with the onset of obesity and eating disorders^7^.

Intermittent access to a HFD reportedly causes binge-like eating behavior in a rodent model, as shown by increases in the motivation to consume^8^ and gradual increases in consumption^9,10^. These behaviors are accompanied by the activation of neurons in the VTA as well as striatum, where an increase in extracellular dopamine concentration has been shown in a rodent model^7,11^. Thus, intermittent access to a HFD is a good model to clarify mechanisms underlying the binge eating of such a diet.

Gamma-aminobutyric acid (GABA), an inhibitory neurotransmitter, acts on two types of receptors: ionotropic GABA_A_ and GABA_C_; and metabotropic GABA_B_ receptors (GABA_B_Rs) that are located both pre- and postsynaptically^12,13^. We previously reported that the GABA_B_R agonist, baclofen, reduced food intake as well as body weight (BW) in obese mice^14^, and BW in obese patients^15^. Baclofen is also effective in reducing binge-like eating in rodents^16–18^ as well as binge eating in humans^19–21^. Our recent study has shown that GABA_B_R signaling in the mesolimbic system is involved in the suppression of the binge-like consumption of a HFD in mice^22^, although the precise sites of action in the system still remain unclear.

In the present study, we employed corticostriatal neuron-specific GABA_B_R-deficient mice, and subjected them to intermittent access to a HFD in order to clarify critical sites of GABA_B_R signaling in the suppression of binge-like consumption with a HFD.

## Results

### Intermittent access to a HFD induces binge-like eating with neural activations in the striatum

**Figure 1:**
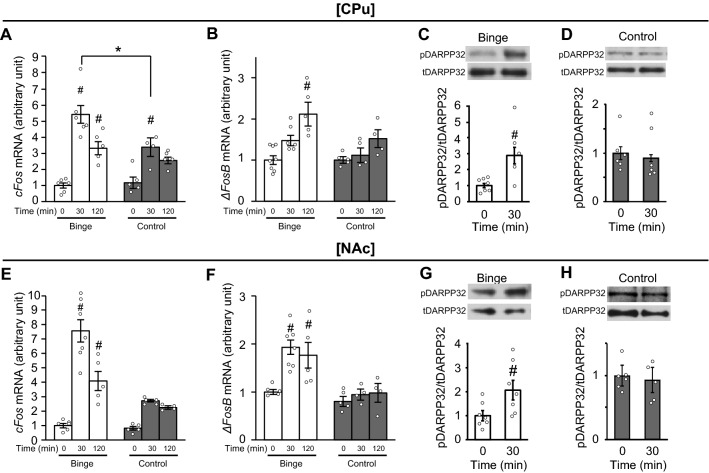
Intermittent access to a HFD induces binge-like eating with neural activation in the striatum. (**A** and **B**) The mRNA expression levels of *cFos* (**A**) and *ΔFosB* (**B**) in the caudate putamen (CPu) in binge and control mice groups. (**C** and **D**) The phosphorylation of dopamine- and cyclic-AMP–regulated phosphoprotein of molecular weight 32,000 (DARPP32) in the CPu in binge (**C**) and control (**D**) groups. (**E** and **F**) The mRNA expression levels of *cFos* (**E**) and *ΔFosB* (**F**) in the nucleus accumbens (NAc) in binge and control groups. (**G** and **H**) The phosphorylation of DARPP32 in the NAc in binge (**G**) and control (**H**) groups. All values are mean ± SEM. Statistical analyses were performed using two-way factorial analysis of variance (ANOVA) (**A**,**B**,**E**, and **F**) followed by Sidak’s post-hoc test or an unpaired *t*-test (**C**,**D**,**G**, and **H**). #*p* < 0.05 versus 0 min in the same group. *p < 0.05 versus control. See also Supplemental Table 2 for details on statistics.

The experimental protocol used is described in Supplemental Fig. 1A. As shown in Supplemental Fig. 1B, BW on the experimental day did not differ between control and binge groups. On the experimental day, mice in the binge group only consumed a HFD over 2 h, with consumed calories higher than in the control group (Supplemental Fig. 1C). We evaluated cFos expression at 30 and 120 min in the limbic system in a binge-like eating model based on previous studies ^10,23^. The mRNA expression level of *cFos* in the CPu was significantly increased 30 and 120 min after the consumption of a HFD compared to values at time 0 in the binge group (Fig. 1A). While the *cFos* mRNA expression level in the CPu was increased at 30 min in the control group, absolute values were lower in the control group compared to the binge group (Fig. 1A). The *cFos* mRNA expression levels in the CPu were proportional to HFD consumption at 30 min in the binge group (Supplemental Fig. 1D). The mRNA expression level of *ΔFosB* in the CPu was increased significantly 120 min after the consumption of a HFD in the binge, but not control group (Fig. 1B). The phosphorylation of dopamine- and cyclic-AMP–regulated phosphoprotein of molecular weight 32,000 (DARPP32), a prominent mediator of dopamine signaling in the striatum^24^, in the CPu was significantly increased at 30 min in the binge group (Fig. 1C), but not in the control group (Fig. 1D). The mRNA expression levels of both *cFos* and *ΔFosB* in the NAc were significantly increased by the consumption of a HFD at 30 and 120 min in the binge, but not control group (Fig. 1E and F). The phosphorylation of DARPP32 was also significantly increased at 30 min in the binge (Fig. 1G), but not control group (Fig. 1H). The *cFos* mRNA expression levels in the PFC were significantly increased at 30 min compared to values at 0 min in both binge and control groups (Supplemental Fig. 1E). The *ΔFosB* mRNA expression levels in the PFC were significantly increased at 120 min compared to values at 0 min in both binge and control groups (Supplemental Fig. 1F). No significant differences in *cFos* and *ΔFosB* mRNA expression levels at all measurement times were found between binge and control groups (Supplemental Fig. 1E and F). The consumption of a HFD for 30 min did not affect dopamine receptor d1 (drd1) expression in CPu and NAc of mice in the binge group (Supplemental Fig. 1G and H).

### GABA_B_R deficiency in corticostriatal neurons increases binge-like eating with activation of drd1-expressing neurons in the CPu

**Figure 2:**
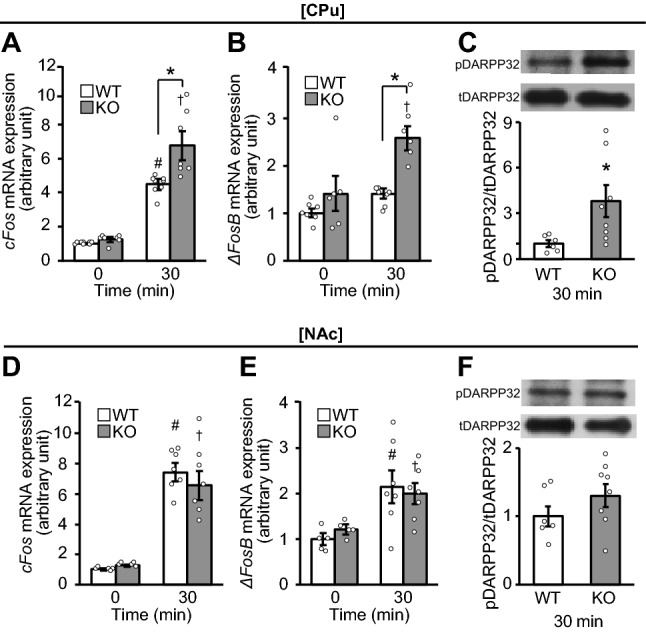
GABA_B_R deficiency in striatum neurons increases expression levels of *cFos* and *ΔFosB* mRNAs as well as phosphorylation of DARPP32 in CPu neurons. (**A**–**C**) The mRNA expression levels of *cFos* (**A**) and *ΔFosB* (**B**), and phosphorylation of dopamine- and cyclic-AMP–regulated phosphoprotein of molecular weight 32,000 (DARPP32) protein (**C**) in the caudate putamen (CPu) in wild-type (WT) and knockout (KO) mice of the binge group. (**D**–**F**) The mRNA expression level of *cFos* (**D**) and *ΔFosB* (**E**), and phosphorylation of DARPP32 (**F**) in the nucleus accumbens (NAc) in WT and KO mice of the binge group. All values are mean ± SEM. Statistical analyses were performed using two-way factorial analysis of variance (ANOVA) (**A**,**B**,**D** and **E**) followed by Sidak’s post-hoc test or unpaired *t*-test (**C** and **F**). #*p* < 0.05 versus 0 min in WT mice. †*p* < 0.05 versus 0 min in KO mice. *p < 0.05 versus WT mice. See also Supplemental Table 2 for details on statistics.

Consumption of a HFD for 30 and 120 min was significantly increased in male knockout (KO) mice compared to WT mice in the binge group (Supplemental Fig. 2A), as reported previously^22^. The mRNA expression levels of both *cFos* and *ΔFosB* in the CPu after the consumption of a HFD were significantly higher at 30 min in corticostriatal neuron-specific GABA_B_R-deficient KO mice compared to wild-type (WT) mice in the binge group (Fig. 2A and B). The phosphorylation of DARPP32 in the CPu was also significantly increased in KO mice compared to WT mice 30 min after the consumption of a HFD (Fig. 2C). In comparison, significant differences were not noted in the mRNA expression levels of *cFos* and *ΔFosB,* and the phosphorylation of DARPP32 in the NAc between genotypes 30 min after the consumption of a HFD (Fig. 2D–F). There were no significant differences in drd1 expression levels in the CPu and NAc between KO and WT mice 30 min after the consumption of a HFD in the binge group (Supplemental Fig. 1B and C).

**Figure 3:**
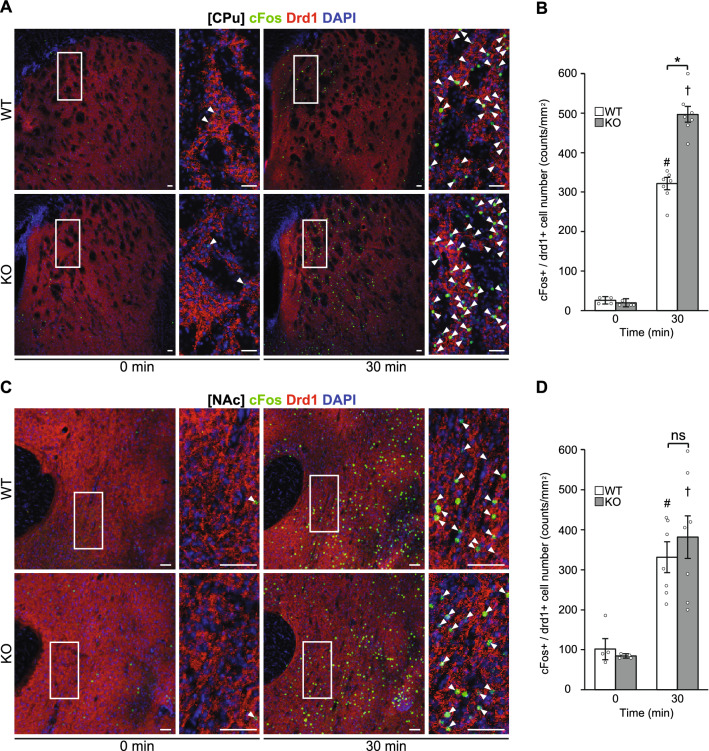
GABA_B_R deficiency in striatum neurons increases expression of cFos + /drd1 + cells in CPu neurons. (**A** and **C**) Representative photographs showing the staining of cFos (green), dopamine receptor d1 (drd1) (red), and DAPI (blue) in the caudate putamen (CPu) (**A**) and nucleus accumbens (NAc) (**C**) of wild-type (WT) and knockout (KO) mice in the binge group. White arrowheads show the colocalization of cFos and drd1. Scale bars: 50 μm. (**B** and **D**) cFos-positive cells in drd1-positive neurons (cFos + /drd1 +) in the CPu (**B**) and NAc (**D**) of WT and KO mice in the binge group. All values are mean ± SEM. Statistical analyses were performed using two-way factorial analysis of variance (ANOVA) followed by Sidak’s post-hoc test (**B** and **D**). ns: not significant. #*p* < 0.05 versus 0 min in WT mice. †*p* < 0.05 versus 0 min in KO mice. *p < 0.05 versus WT mice. See also Supplemental Table 2 for details on statistics.

Immunohistochemistry in the striatum showed that while cFos-positive cells in drd1-positive neurons (cFos + /drd1 +) in the CPu were significantly increased 30 min after the consumption of a HFD in both WT and KO mice in the binge group, the numbers were significantly greater in KO than in WT mice (Fig. 3A and B). In contrast, no significant differences were observed in the number of cFos-positive cells in drd1-positive neurons (cFos + / drd1 +) in the NAc between genotypes (Fig. 3C and D). Significant differences in the number of cFos-positive cells in drd1-negative neurons (cFos + / drd1−) in the CPu and NAc were not found between genotypes (Supplemental Fig. 3A and B).

### Baclofen suppresses binge-like eating and neural activation in the CPu

**Figure 4:**
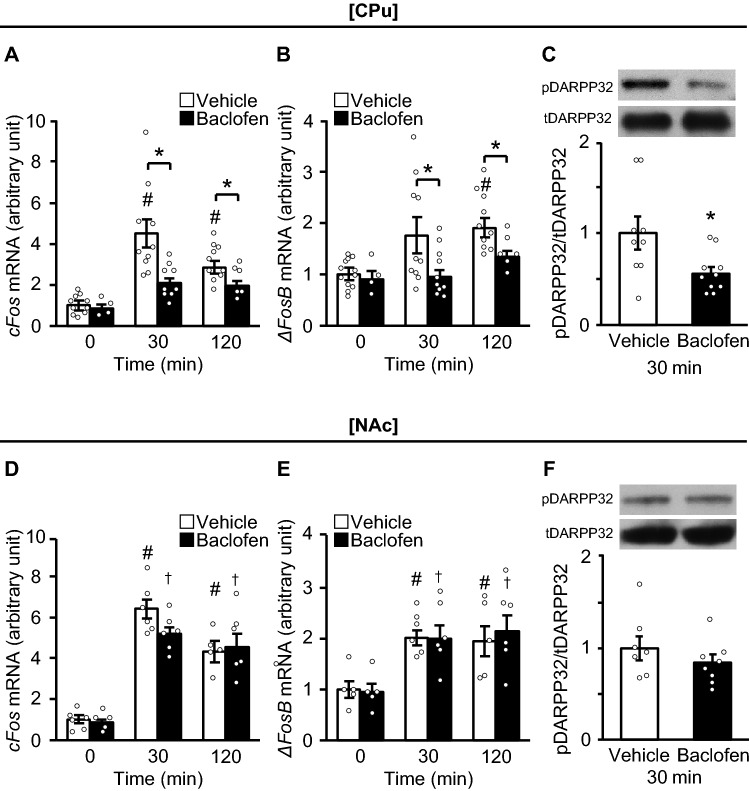
Baclofen suppresses *cFos* and *ΔFosB* mRNA expression levels as well as phosphorylation of DARPP32 in the CPu. (**A**–**C**) The mRNA expression levels of *cFos* (**A**) and *ΔFosB* (**B**), and phosphorylation of dopamine- and cyclic-AMP–regulated phosphoprotein of molecular weight 32,000 (DARPP32) protein (**C**) in the caudate putamen (CPu) in wild-type (WT) mice of the binge group treated with baclofen and vehicle. (**D**–**F**) The mRNA expression levels of *cFos* (**D**) and *ΔFosB* (**E**), and phosphorylation of DARPP32 (**F**) in the nucleus accumbens (NAc) in WT mice of the binge group treated with baclofen and vehicle. All values are mean ± SEM. Statistical analyses were performed using two-way factorial analysis of variance (ANOVA) (**A**,**B**,**D** and **E**) followed by Sidak’s post-hoc test or an unpaired *t* test (C and F). #*p* < 0.05 versus 0 min in WT mice. †*p* < 0.05 versus 0 min in KO mice. *p < 0.05 versus WT mice. See also Supplemental Table 2 for details on statistics.

As reported previously^22^, treatment with baclofen, a GABA_B_R agonist, significantly reduced HFD consumption compared to vehicle in WT mice, but not in KO mice (Supplemental Fig. 4A). Baclofen suppressed elevation of *cFos* and *ΔFosB* mRNA expression levels in the CPu compared to vehicle at 30 and 120 min after the consumption of a HFD (Fig. 4A and B). The phosphorylation of DARPP32 was also significantly suppressed by baclofen compared to vehicle (Fig. 4C). In contrast, baclofen did not affect the mRNA expression levels of *cFos* or *ΔFosB,* or the phosphorylation of DARPP32 in the NAc, after the consumption of a HFD (Fig. 4D–F).

### Suppressive effects of baclofen on activation of drd1-expressing neurons in the CPu were absent in KO mice

**Figure 5:**
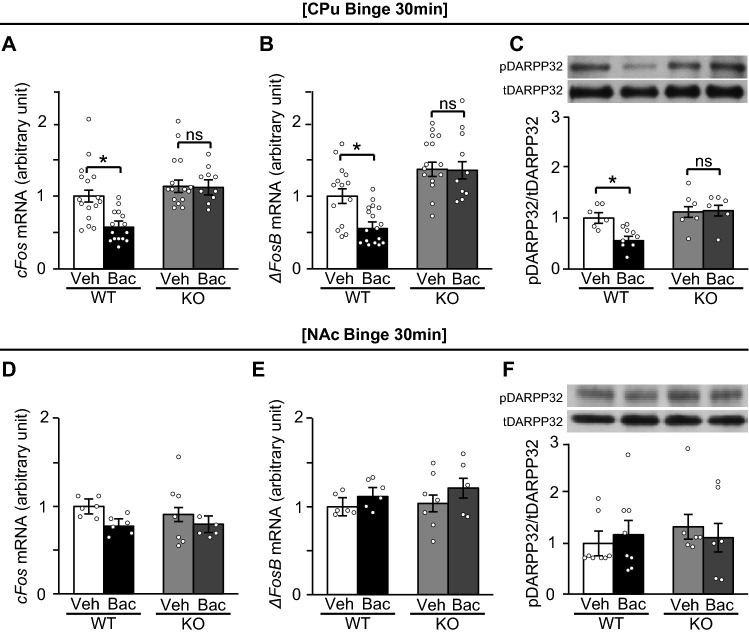
Suppressive effects of baclofen on *cFos* and *ΔFosB* mRNA expression levels as well as phosphorylation of DARPP32 in the CPu were absent in KO mice. (**A**–**C**) The mRNA expression levels of *cFos* (A) and *ΔFosB* (**B**), and phosphorylation of dopamine- and cyclic-AMP–regulated phosphoprotein of molecular weight 32,000 (DARPP32) protein (**C**) in the caudate putamen (CPu) in wild-type (WT) and knockout (KO) mice of a binge group on a high fat diet (HFD) treated with vehicle and baclofen. (**D**–**F**) The mRNA expression levels of *cFos* (**D**) and *ΔFosB* (**E**), and phosphorylation of DARPP32 (**F**) in the nucleus accumbens (NAc) in WT and KO mice of a binge group treated with baclofen and vehicle. All values are mean ± SEM. Statistical analyses were performed using two-way factorial analysis of variance (ANOVA) followed by Sidak’s post-hoc test (**A**–**F**). ns: not significant, veh: vehicle, bac: baclofen. *p < 0.05 versus vehicle in WT mice. See also Supplemental Table 2 for details on statistics.

The suppressive effects of baclofen on *cFos* and *ΔFosB* mRNA expression levels, as well as the phosphorylation of DARPP32 in the CPu, were absent in KO mice in the binge group (Fig. 5A–C). Baclofen also did not affect the mRNA expression levels of *cFos* and *ΔFosB,* and the phosphorylation of DARPP32 in the NAc in both WT and KO mice (Fig. 5D–F).

**Figure 6:**
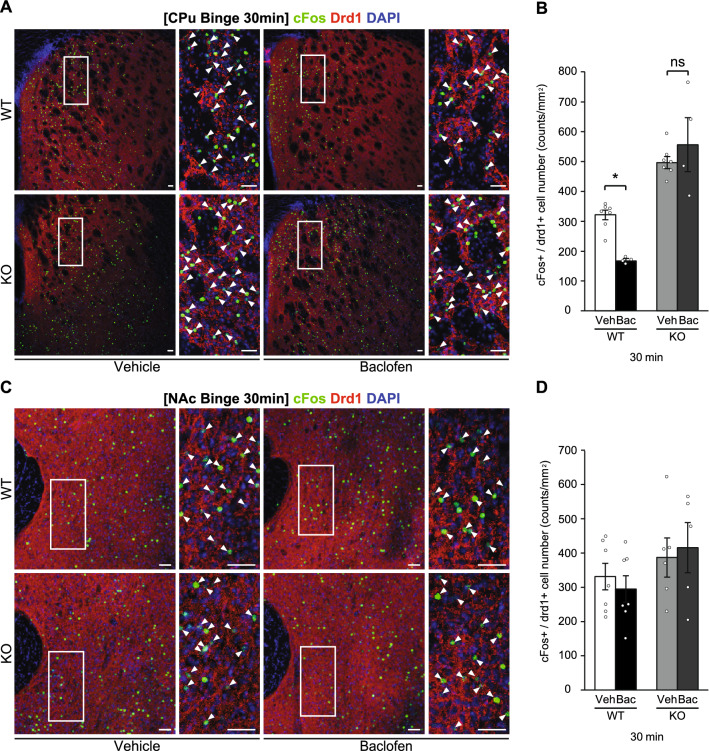
Suppressive effects of baclofen on cFos + /drd1 + cell expression in the CPu were absent in KO mice. (**A** and **C**) Representative photographs showing the staining of cFos (green), dopamine receptor d1 (drd1) (red), and DAPI (blue) in the caudate putamen (CPu) (**A**) and nucleus accumbens (NAc) (**C**) of wild-type (WT) and knockout (KO) mice at 30 min on a high fat diet (HFD) in the binge group. White arrowheads show the colocalization of cFos and drd1. Scale bars: 50 μm. (**B** and **D**) cFos-positive cells in drd1-positive neurons (cFos + /drd1 +) in the CPu (**B**) and NAc (**D**) of WT and KO mice at 30 min on a HFD in the binge group. All values are mean ± SEM. Statistical analyses were performed using two-way factorial analysis of variance (ANOVA) followed by Sidak’s post-hoc test (**B** and **D**). ns: not significant, veh: vehicle, bac: baclofen. *p < 0.05 versus vehicle in WT mice. See also Supplemental Table 2 for details on statistics.

Immunohistochemical staining in the striatum showed that baclofen significantly reduced the number of cFos-positive cells in drd1-positive neurons (cFos + /drd1 +) in the CPu 30 min after the consumption of a HFD in WT, but not in KO mice (Fig. 6A and B). Significant differences were not found in the number of cFos-positive cells in drd1-positive neurons (cFos + /drd1 +) in the NAc between vehicle and baclofen groups in both WT and KO mice (Fig. 6C and D). Also, no significant differences were evident in the numbers of cFos-positive cells in drd1-negative neurons (cFos + /drd1−) in the CPu and NAc between vehicle and baclofen groups in both WT and KO mice (Supplemental Fig. 5A and B).

## Discussion

In the present study, we showed that drd1-expressing neurons in the CPu and NAc were significantly activated in WT mice after binge-like consumption on a HFD. Our data also showed that activation of drd1-expressing neurons was significantly enhanced in the CPu, but not in the NAc, in corticostriatal neuron-specific GABA_B_R-deficient mice compared to WT mice after binge-like consumption on a HFD. Furthermore, the GABA_B_R agonist, baclofen, suppressed the activation of neurons expressing drd1 in the CPu and decreased binge-like eating by WT mice on a HFD, but not that of KO mice. These data suggest GABA_B_R signaling in the CPu is crucial for the suppression of binge-like eating while on a HFD.

Previous studies using binge-like eating models in rats showed that the number of c-Fos–positive cells in the CPu, as well as ΔFosB expression and DARPP32 phosphorylation in the NAc, were increased after the intake of palatable food^9,25,26^. These data are consistent with the findings in the current study that neurons in the CPu and NAc were activated in WT mice with intermittent access to a HFD. Previous studies also showed that the dorsal medial PFC is associated with binge-like eating^23^, whereas the ventral medial PFC is associated with resistance to binge-like eating^27^. While our data did not reveal any difference in dopamine receptor signaling in the PFC between binge and control groups, our analyses of whole PFC do not exclude the role of PFC in binge-like eating.

We previously reported that GABA_B_R signaling in the mesolimbic system is involved in the suppression of binge-like consumption under a HFD^22^; this was confirmed in the current study showing that binge-like eating during a HFD was enhanced in corticostriatal neuron-specific GABA_B_R-deficient mice. In addition, we clearly demonstrated that the suppression of binge-like eating during a HFD by baclofen was accompanied by decreased activity of drd1-expressing neurons in the CPu, but not in the NAc. The site of activation in the striatum, reportedly, depended on the type of food or drink ingested: ethanol (EtOH) infusion, but not fat intake, significantly increased NAc dopamine signaling, while fat intake, but not EtOH infusion, significantly increased CPu dopamine signaling^28^. It was also shown that taste quality regulated dopamine release in the NAc, while dopamine in the CPu was released when a nutritive solution, but not a non-nutritive solution, was ingested^29^. Taken together with our findings, it is suggested that the CPu plays an important role in regulating the binge-like consumption of calorie-enrich palatable food such as when on a HFD, where GABA_B_R signaling suppresses the activity of drd1-expressing neurons.

**Figure 7:**
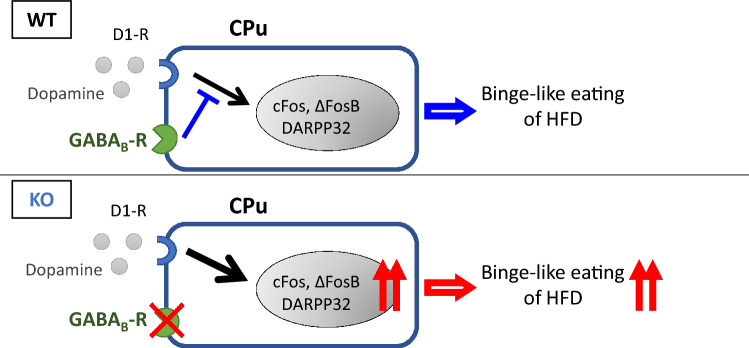
Schematic of role of GABA_B_R signaling in striatum in suppression of binge-like consumption of a HFD. We suggest that gamma-aminobutyric acid type B receptor (GABA_B_R) signaling in the caudate putamen (CPu) suppresses the binge-like consumption of a high fat diet (HFD) by inhibiting downstream of dopamine receptor d1 (drd1) signaling in the CPu. The figure image was drawn using PowerPoint 2017 (Microsoft, USA).

Our data showed that the consumption of a HFD did not affect drd1 expression levels in the CPu, and that these levels did not differ between WT and KO mice. Taken together with the findings that a GABA_B_R agonist suppressed mRNA expression levels of cFos and ΔFosB as well as the phosphorylation of DARPP32 in the CPu in WT mice, and that they were significantly increased in KO mice compared to WT mice, it is suggested that GABA_B_R signaling suppressed signaling downstream of drd1 in the CPu (Fig. 7).

The first limitation in the present study is that we cannot rule out the possibility that GABA_B_R signaling in the medial PFC and orbitofrontal cortex (OFC), which are important sites for decision making and related to reward-based behavior^30,31^, might be affected in KO mice, since GPR88-positive neurons include not only striatal neurons but also those in the medial PFC and OFC^22^. Second, we did not measure concentrations of dopamine in the striatum but used surrogate markers for dopamine signaling. Third, the systemic injection of baclofen was employed in the current study. To clarify the site of action, a local infusion of baclofen would be warranted in future studies. Fourth, while our study focused on dopamine receptor d1 signaling, this does not exclude the possibility that dopamine receptor d2 signaling in the CPu and NAc might also be involved in binge-like consumption that occurs during a HFD.

In conclusion, our data suggest that GABA_B_R signaling in CPu neurons expressing drd1 suppresses binge-like consumption during a HFD in mice.

## Methods

### Mice

All experiments were performed using 9-week-old male mice. All animal procedures were approved by the Animal Care and Use Committee of Nagoya University Graduate School of Medicine and performed in accordance with National Institutes of Health animal care guidelines. The study was carried out in compliance with ARRIVE guidelines. Mice were maintained on a 12 h light/12 h dark cycle (lights on from 09:00 to 21:00) in a temperature-controlled barrier facility, with free access to water and food. Age-matched littermates were used for all experiments.

### Mice with GPR88-sepecific deletion of GABA_B1_ receptor

We crossed *GABA*_*B1*_*R*^*lox511*^^*/lox511*^ mice with *GPR88-Cre* heterozygous mice to generate *GABA*_*B1*_*R*^*lox511/lox511*^* GPR88-Cre* mice (KO mice)*, GABAB*^+*/lox511*^* GPR88-Cre* mice*,* and *GABA*_*B1*_*R*^*lox511/lox511*^ littermate controls (WT mice). *GABA*_*B1*_*R*^*lox511/lox511*^ mice have been generated previously^32^. *GPR88-Cre* transgene (RRID: IMSR_RBRC10287) mice express functional Cre-recombinase mainly in medium spiny neurons and a small population of parvalbumin-positive interneurons in the CPu and NAc^32^. *GPR88-Cre* transgene mice were provided by RIKEN BRC (Koyodai, Japan) through the National Bio-Resource Project of the Ministry of Education, Culture, Sports, Science and Technology, Japan. DNA was extracted from the tail of each experimental mouse at the age of 10 days. DNA was subjected to genotyping analyses by PCR with KOD FX DNA polymerase (Toyobo, Osaka, Japan) and oligonucleotide primers. The PCR was performed with a SimpliAmp™ Thermal Cycler (Applied Biosystems™, Foster City, CA, USA). The conditions were 5 min at 95 °C followed by 30 cycles at 95 °C for 30 s, 56 °C for 20 s, and 72 °C for 60 s with a 7 min final extension. Primer sequences used for genotyping of *GABA*_*B1*_*R*^*lox511/lox511*^ and *GPR88-Cre* mice were as follows: *GABA*_*B1*_*R* forward, 5’-TGGGGTGTGTCCTACATGCAGCGGACGG; reverse, 5’-GCTCTTCACCTTTCAACCCAGCCTCAGGC AGGC; *GPR88-Cre* forward, 5’- ACC TGATGGACATGTTCAGGGATCG; reverse, 5’-TCCGGTTATTCAACTTGCACCATGC. All *GABA*_*B1*_*R*^*lox511/lox511*^ mice and *GPR88-Cre* mice were backcrossed more than 10 generations onto a C57BL/6 J background.

### Training and assessment of binge-like eating behavior

We used a published protocol^22^ to train and assess binge-like eating behavior in male mice (Supplemental Fig. 1A). After acclimation, WT mice (9 weeks of age) were exposed to both a chow diet (CD; CE-2, CLEA Japan, Tokyo, Japan; 24.9% protein, 4.6% fat, and 70.5% carbohydrate) and a HFD (Test Diet 58Y1; PMI Nutrition International, KS, USA; 18.3% protein, 60.9% fat, and 20.1% carbohydrate) for 2 days, and then exposed to only CD for 5 days. Next, the mice were divided into two groups: ‘‘Binge group’’ and ‘‘Control group’’, on the experimental day. The mice in the binge group were given free access to HFD and CD for 120 min (zeitgeber time [ZT] 12 to 14: 21:00 to 23:00), while the mice in the control group were continuously fed only CD. Then HFD and CD intakes were measured for 120 min (ZT 12 to 14). The food intake of mice on both the CD and HFD were assessed by multifeeders (Shinfactory, Fukuoka, Japan). Mice in the binge group were sacrificed at 30 and 120 min after the start of access to HFD and CD (i.e., 21:30 and 23:00, respectively) and those in the control group were also sacrificed at 21:30 and 23:00. The GABA_B_R agonist, baclofen (Sigma–Aldrich, St Louis, MO, USA), dissolved in 0.9% saline, or vehicle (saline) were administered intraperitoneally 30 min before the beginning of the dark cycle (ZT 12) on the experimental day. The dose of baclofen was 3 μg/10 μL/g body weight.

### Extraction of brain tissues

After mice were sacrificed, the CPu, NAc, and PFC were rapidly dissected from 2.0-mm thick coronal sections of fresh brain using an Alto Stainless Steel Coronal 1.0 mm Brain Matrix (Roboz Surgical Instrument Co., MD, USA) and Sharp Matrix Blades (Kent Scientific Co., Torrington, CT, USA). The NAc was punched out using 1.5-mm Miltex Disposable Biopsy Punches (Integra Life Science, West Valley City, NJ, USA) with the anterior commissure in the center of the circle. The CPu and PFC were dissected using tweezers (MA-65, Natsume Seisakusho Co., Tokyo, Japan) ^33^. The dissected tissues were immediately frozen in liquid nitrogen until RNA extraction.

### Determination of mRNA levels by qRT–PCR

Total RNA was extracted from samples using TRIzol (Invitrogen, Carlsbad, CA, USA) and an RNeasy kit (QIAGEN, Hilden, Germany). Copy DNA was synthesized from 60 to 150 ng total RNA using a ReverTra Ace qPCR RT Kit (TOYOBO, Osaka, Japan). Quantitative reverse transcriptase (qRT)–PCR reactions were carried out using Brilliant III Ultra-Fast SYBR Green QPCR Master Mix (Agilent Technologies, Santa Clara, CA, USA), and samples were processed using a CFX Connect Real-Time PCR Detection System (Bio-Rad Laboratories, Hercules, CA, USA). The relative mRNA levels of *cFos* and *ΔFosB* were assessed by qRT–PCR using glyceraldehyde 3-phosphate dehydrogenase (*Gapdh*) as an internal control. The qRT–PCR reactions were carried out, and relative mRNA expression levels were calculated using a comparative Ct method as described previously^34,35^. The sequences of primers are described in Supplemental Table 1.

### Determination of protein levels by western blot

Samples of CPu or NAc were lysed in a buffer (100 μL) containing Tris (10 mM, pH 7.4), NaF (50 mM), NaCl (150 mM), SDS (0.1%), Na_3_VO_4_ (2 mM), EDTA (5 mM), Triton X-100 (1%, Sigma–Aldrich) sodium deoxycholate (minimum 1%), and a protease inhibitor mix (1%; Roche, Stockholm, Sweden). After centrifuging samples, protein concentrations in the supernatants were determined using a bicinchoninic acid kit (Sigma–Aldrich). Protein (7.5–10 μg per sample) samples were run on a Bis–Tris gel (10%; Invitrogen) and transferred onto polyvinylidene fluoride membranes (Millipore, Burlington, MA, USA). Blots were blocked for 1 h in Blocking One, Blocking One P (Nacalai Tesque Inc., Kyoto, Japan) or in TBS-T solution [10 mM Tris–HCl (pH 7.4), 150 mM NaCl, and 0.1% Tween] containing 5% skim milk. Membranes were incubated overnight at 4 °C with the following antibodies: rabbit anti-DARPP32 phosphorylated at Thr34 (#12,438; Cell Signaling Technology, Danvers, MA, USA, RRID: AB_2797914) and mouse anti-dopamine D1R/DRD1 (clone SG2-D_1a_; #NB110-60,017; Novus Biologicals, Briarwood Avenue, USA, RRID: AB_905382). Primary antibodies were probed with horse radish peroxidase–conjugated donkey anti-rabbit IgG (NA934; GE Healthcare, Little Chalfont, UK, RRID: AB_772206) and horse radish peroxidase–conjugated sheep anti-mouse IgG (NA931; GE Healthcare, Little Chalfont, UK, RRID: AB_772210) for 1 h at room temperature. To improve sensitivity and the signal-to-noise ratio, Can Get Signal Immunoreaction Enhancer Solution (TOYOBO, Osaka, Japan) was used for the dilution of primary and secondary antibodies. Immunoreactivity was detected using ECL Prime Western Blotting Detection Reagent (GE Healthcare, Chicago, IL, USA). The membranes were stripped and incubated with rabbit antibodies against nonphosphorylated DARPP32 antibody (ab40801; Abcam, Cambridge, UK, RRID: AB_731843) and rabbit anti-beta actin antibody (ab8227; Abcam, Cambridge, UK, RRID: AB_2305186) for normalization. Full immunoblots are displayed in Supplemental Fig. 6.

### Brain collection for immunohistochemistry

Male mice were deeply anesthetized using 2.0% isoflurane with an animal anesthetizer device (MK-AT210; Muromachi Kikai, Tokyo, Japan) and transcardially perfused with a cold fixative containing paraformaldehyde (4%; PFA) in phosphate buffered saline (PBS) pH 7.4, between ZT 12 and ZT 12:30 in the fed state. After fixation, brains were removed and left in the same fixative overnight at 4 °C. Then, brains were cut into 50-µm coronal sections using a vibratome (VT1200S; Leica Co, Wetzlar, Germany) and stored at −30 °C using cryoprotective solution until immunohistochemistry.

### Immunohistochemistry

Free-floating sections were blocked with Triton X-100 (0.3%; 30 min) and bovine serum albumin solution (5%; 1 h) at room temperature. They were then incubated in diluted blocking buffer (Blocking one, NACALAI TESQUE, Japan) and 0.2% sodium azide for 5 days at 4 °C with the following antibodies: rabbit anti-cFos (226 003, Synaptic Systems, Goettingen, Germany, RRID: AB_2231974), and mouse anti-drd1 (sc-33660, Santa Cruz Biotechnology, Dallas, TX, USA, RRID: AB_668813). The sections were then incubated with Tween-20 (0.05%; 40 min) followed by Alexa Fluor 488-conjugated anti-rabbit IgG secondary antibody (Invitrogen; RRID: AB_221544), Alexa Fluor 594 conjugated anti-mouse IgG secondary antibody (Invitrogen; RRID: AB_2650601), and DAPI (D523; DOJINDO, Kumamoto, Japan) for 2 h at room temperature. After washing in Tween-20 (0.05%), sections were placed on slides, air dried, and Vectashield (Vector Labs, Peterborough, UK) used to fix coverslips in place. All fluorescently stained sections were examined with either a confocal laser microscope (TiEA1R; NIKON INSTECH, Tokyo, Japan) or a fluorescence microscope (BZ-9000; Keyence, Osaka, Japan; RRID:SCR_015486), and viewed using NIS-Elements software (NIKON INSTECH; RRID:SCR_014329). Cells labeled for cFos colocalization, with or without drd1, were counted bilaterally in a blinded fashion. cFos was merged with nuclear marker (DAPI), while drd1 was expressed in the cytoplasm. For analysis, samples from 4–10 mice from each group were used for staining and the mean values from two or three serial sections from each mouse were calculated. The sections included the CPu and NAc, which were located 1.10–1.42 mm from the bregma based on coordinates in a mouse brain atlas. The anatomical boundaries of each brain region were also identified using the mouse brain atlas^37^.

### Statistical analysis

The statistical significance of differences between groups was analyzed by either unpaired *t-*test, two-way factorial analysis of variance (ANOVA) or two-way ANOVA assessed by repeated measures followed by Sidak’s multiple comparison by using SPSS Statistics 26 (IBM, Endicott, NY, USA; RRID:SCR_002865). Results are expressed as mean ± standard error of the mean (SEM), and differences were considered significant at *p* < 0.05.

## Supplementary Information

Supplementary Information.

## Data Availability

The datasets generated and/or analyzed during the current study are available from the corresponding author on reasonable request.
