# Supplementary material for: GABAB receptor signaling in the caudate putamen is involved in binge-like consumption during a high fat diet in mice
Source: Sci Rep. 2021 Sep 29;11:19296. doi: 10.1038/s41598-021-98590-9 (PMC8481241; doi:10.1038/s41598-021-98590-9)
Supplement: Supplementary file 1 — Supplementary Information. [file 41598_2021_98590_MOESM1_ESM.pdf]

GABA$_B$ receptor signaling in the caudate putamen is involved in binge-like consumption during a high fat diet in mice

[1]Runan Sun (sunrunan1995@med.nagoya-u.ac.jp)

[1,2,*]Taku Tsunekawa (tsune-ta@med.nagoya-u.ac.jp)

[1]Tomonori Hirose (hirose-tomonori@med.nagoya-u.ac.jp)

[1]Hiroshi Yaginuma (h-yaginuma@med.nagoya-u.ac.jp)

[1]Keigo Taki (k-taki@med.nagoya-u.ac.jp)

[1,2]Akira Mizoguchi (mizoguchi.akira@med.nagoya-u.ac.jp)

[1]Takashi Miyata (miyata.takashi@med.nagoya-u.ac.jp)

[1]Tomoko Kobayashi (kobayashi.tomoko@med.nagoya-u.ac.jp)

[1]Mariko Sugiyama (sugiyama.mariko@med.nagoya-u.ac.jp)

[1]Takeshi Onoue (t-onoue@med.nagoya-u.ac.jp)

[1]Hiroshi Takagi (htakagi@med.nagoya-u.ac.jp)

[1]Daisuke Hagiwara (d-hagiwara@med.nagoya-u.ac.jp)

[1]Yoshihiro Ito (yoshi716@med.nagoya-u.ac.jp)

[1]Shintaro Iwama (siwama@med.nagoya-u.ac.jp)

[1]Hidetaka Suga (sugahide@med.nagoya-u.ac.jp)

[1,3]Ryoichi Banno (ryouichi@med.nagoya-u.ac.jp)

[4]Bernhard Bettler (bernhard.bettler@unibas.ch)

[1,#,*]Hiroshi Arima (arima105@med.nagoya-u.ac.jp)

[1]Department of Endocrinology and Diabetes, Nagoya University Graduate School of Medicine, 65 Tsurumai-cho, Showa-ku, Nagoya, 466-8560, Japan

[2]Department of Endocrinology and Diabetes, Ichinomiya Municipal Hospital, 2-2-22, Bunkyo, Ichinomiya, 491-8558, Japan

[3]Research Center of Health, Physical Fitness and Sports, Nagoya University, Nagoya, 464-8601, Japan

[4]Department of Biomedicine, University of Basel, Basel CH-4056, Switzerland

[#]Lead Contact

[*]Authors for correspondence:

Department of Endocrinology and Diabetes, Nagoya University Graduate School of Medicine, 65 Tsurumai-cho, Showa-ku, Nagoya 466-8560,

Japan. Phone: +81-52-744-2142, Fax: +81-52-744-2206

Email: arima105med.nagoya-u.ac.jp (HA)

tsune-ta@med.nagoya-u.ac.jp (TT)

Supplemental Figure 1.

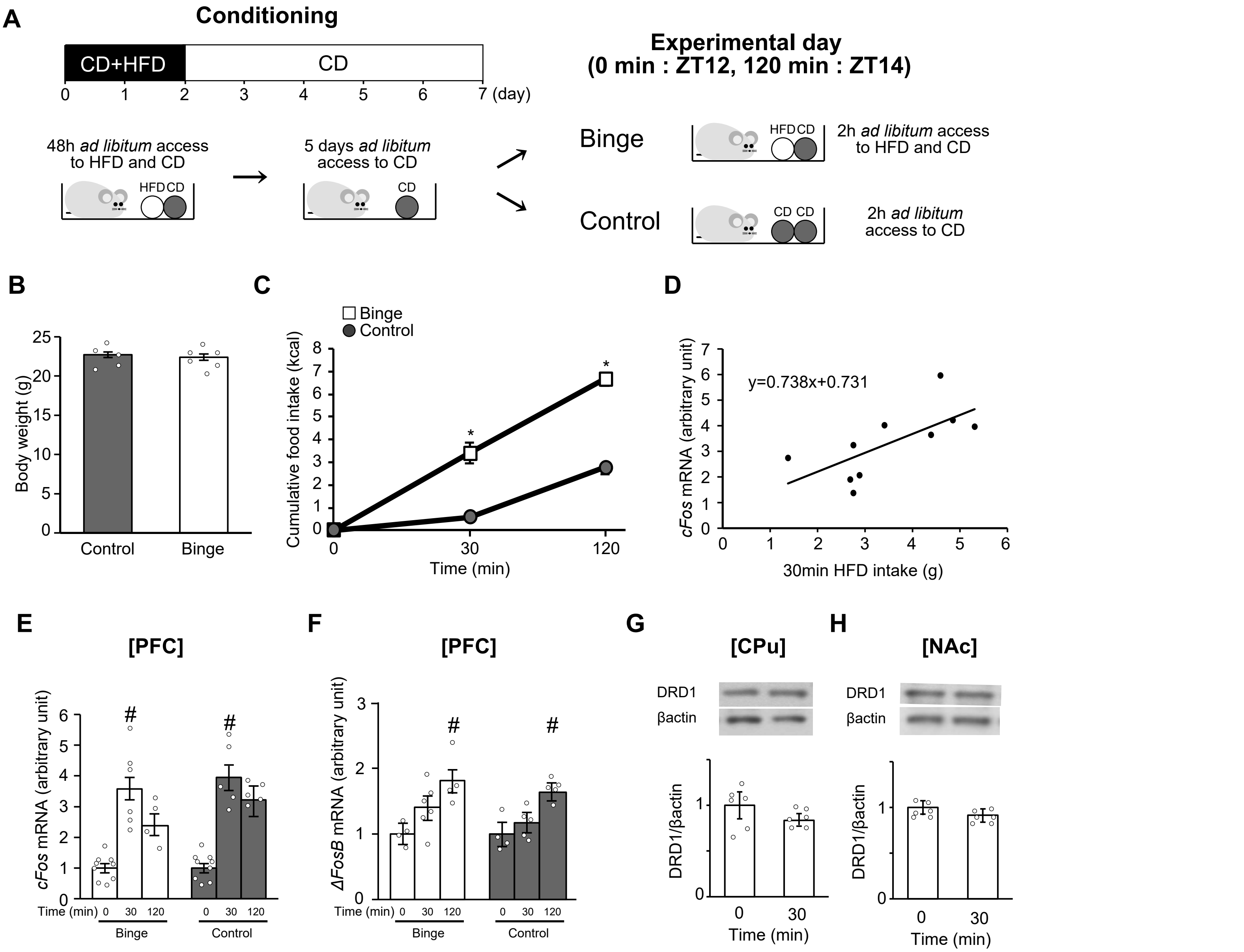

Supplemental Figure 1. Intermittent access to a HFD induces binge-like eating with activation of neurons in the striatum related to Figure 1.
(A) Diagram of the experimental protocol for this study. The figure image was drawn using PowerPoint 2017 (Microsoft, USA).(B) Body weights of wild-type (WT) mice in cont and binge groups at baseline (zeitgeber time [ZT] 12) of each experimental day. (C) Food intake of mice on a chow diet (CD) in the control group and a high fat diet (HFD) in the binge group during zeitgeber times 12 to 14 (ZT12, ZT14) in WT mice. (D) Relationship of cFos and HFD consumption at 30 min in WT mice of the binge group. (E and F) The mRNA expression levels of cFos (E) and ΔFosB (F) in the prefrontal cortex (PFC) of mice in binge and control groups. (G and H) The dopamine receptor d1 (drd1) expression in the caudate putamen (CPu) (G) and nucleus accumbens (NAc) (H) of mice in the binge group. All values are mean ± SEM. Statistical analyses were performed using Pearson's correlation coefficient (D), an unpaired t-test (B, G and H), two-way factorial analysis of variance (ANOVA) assessed by repeated measures (C), or two-way factorial ANOVA (E and F) followed by Sidak's post-hoc test. *$p < 0.05$ versus control. #$p < 0.05$ versus 0 min in WT mice. See also Supplemental Table 2 for details on statistics.

Supplemental Figure 2.

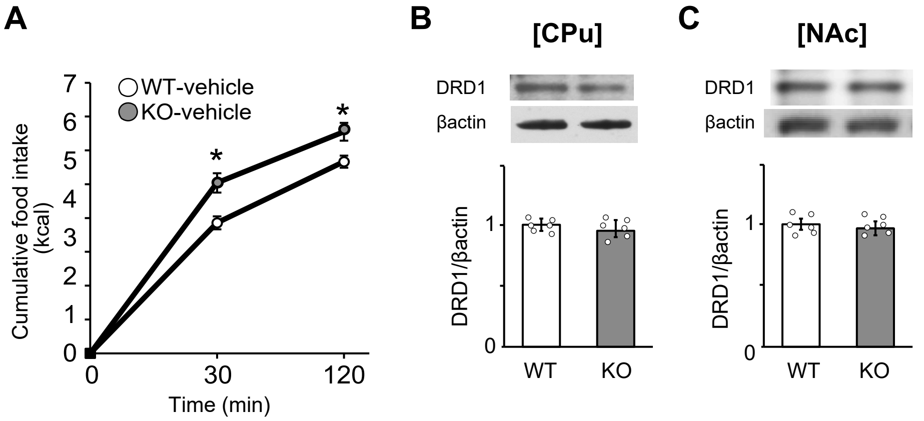

Supplemental Figure 2. GABABR deficiency in striatum neurons increases binge-like eating with neural activation in the CPu related to Figure 2.

(A) Food intake of wild-type (WT) and knockout (KO) mice on a high fat diet (HFD) in the binge group dur zeitgeber times (ZT) 12 to 14. (B and C) The dopamine receptor d1 (drd1) expression in the caudate putamen (CPu) (B) and nucleus accumbens (NAc) (C) in WT and KO mice at 30 min on a HFD in the binge group. All values are mean ± SEM. Statistical analyses were performed using two-way factorial analysis of variance (ANOVA) assessed by repeated measures followed by Sidak's post-hoc test (A) or an unpaired t-test (B and C). *$p < 0.05$ versus WT mice. See also Supplemental Table 2 for details on statistics. GABABR, gamma-aminobutyric acid type B receptor

Supplemental Figure 3.

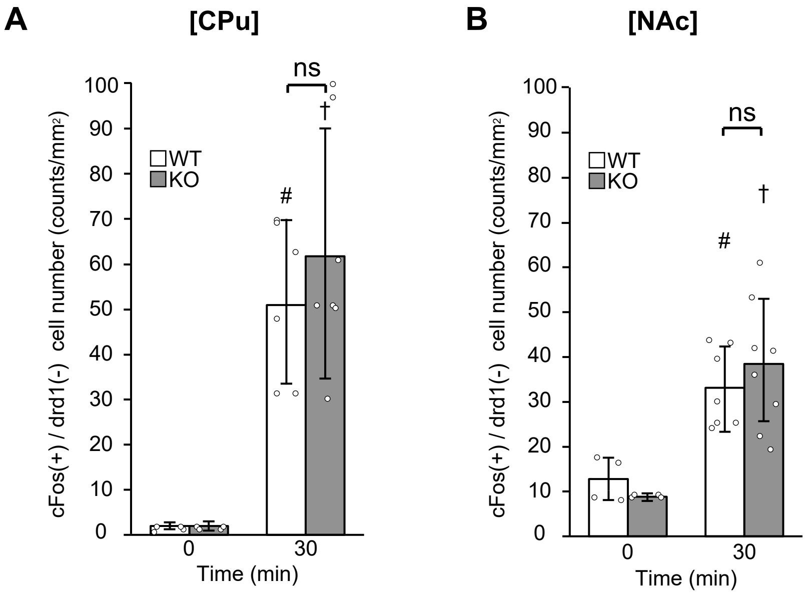

Supplemental Figure 3. GABABR deficiency in striatum neurons has no effect on expression of cFos +/drd1- cells in CPu neurons related to Figure 3.

(A and B) cFos-positive cells in dopamine receptor d1 (drd1)-negative neurons (cFos+/drd1-) in the caudate putamen (CPu) (A) and nucleus accumbens (NAc) (B) of wild-type (WT) and knockout (KO) mice in the binge group. All values are mean ± SEM. The statistical analysis was performed using two-way factorial analysis of variance (ANOVA) followed by Sidak's post-hoc test (A and B). ns: not significant. #p < 0.05 versus 0 min in WT mice. †p < 0.05 versus 0 min in KO mice. See also Supplemental Table 2 for details on statistics. GABABR, gamma-aminobutyric acid type B receptor

Supplemental Figure 4.

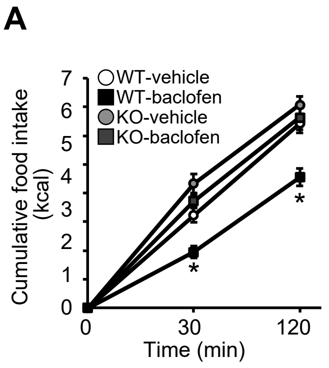

Supplemental Figure 4. Baclofen suppresses binge-like eating during a HFD with inhibition of GABABR signaling in the CPu related to Figure 4.

Intake of a high fat diet (HFD) during zeitgeber times [ZT] 12 to 14 in wild-type (WT) and knockout (KO) mice in the binge group treated with baclofen and vehicle. All values are mean ± SEM. The statistical analysis was performed using two-way factorial analysis of variance (ANOVA) assessed by repeated measures followed by Sidak's post-hoc test. *$p < 0.05$ versus control. See also Supplemental Table 2 for details on statistics. GABABR, gamma-aminobutyric acid type B receptor

Supplemental Figure 5.

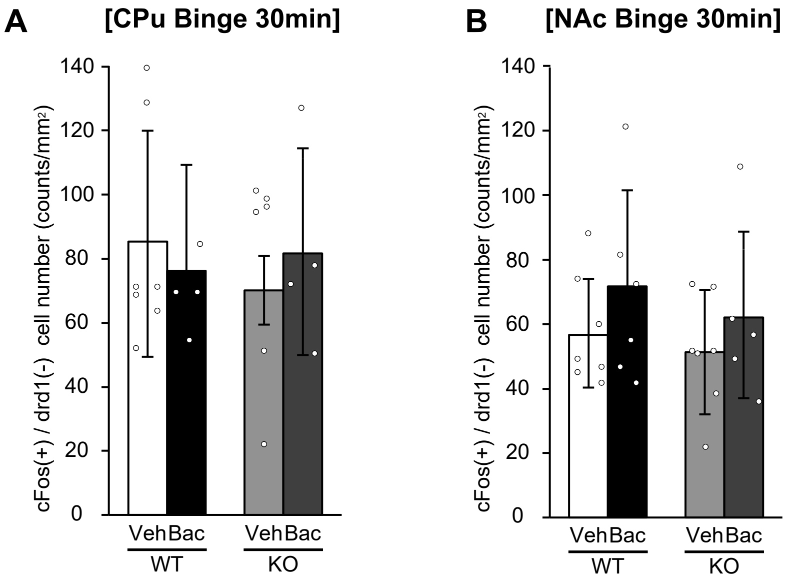

Supplemental Figure 5. Baclofen has no effect on cFos+/drd1- cell expression in the CPu in both WT and KO mice related to Figure 6.

(A and B) cFos-positive cells in dopamine receptor d1 (drd1)-negative neurons (cFos+/drd1-) in the caudate putamen (CPu) (A) and nucleus accumbens (NAc) (B) of wild-type (WT) and knockout (KO) mice at 30 min on a HFD in the binge group. All values are mean ± SEM. Statistical analyses were performed using two-way factorial analysis of variance (ANOVA) followed by Sidak’s post-hoc test (A and B). veh: vehicle, bac: baclofen. See also Supplemental Table 2 for details on statistics.

Supplemental Figure 6. Whole western immunoblots.

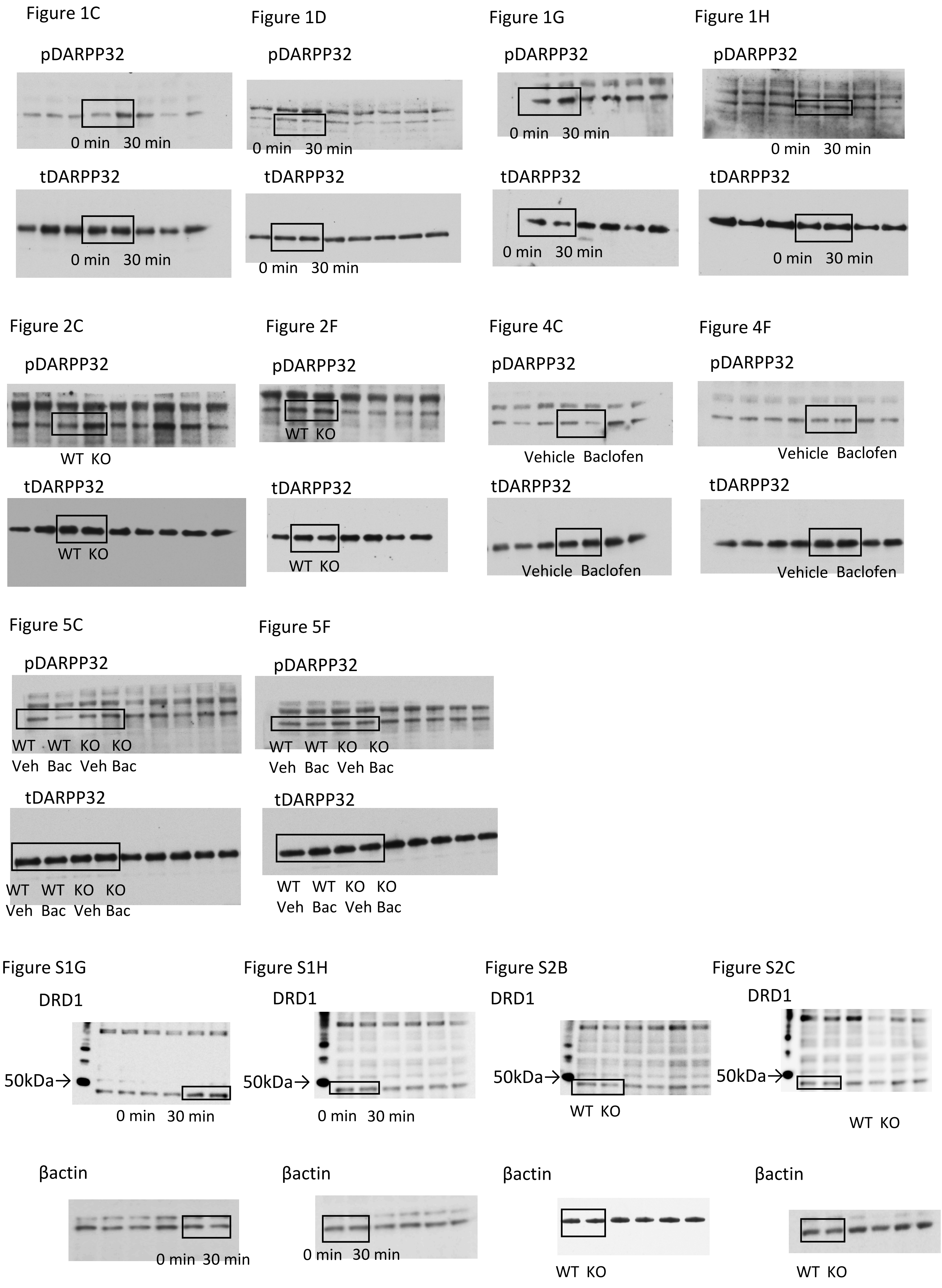

Supplemental Table 1. List of qRT–PCR primers used in this study.

Gene	Common abbreviation	Forward primer (5'→3')	Reverse primer (5'→3')
Fos	cFos	CTGTCAACACACAGGACTTTT	AGGAGATAGCTGCTCTACTTTG
Fosb	ΔFosB	AGGCAGAGCTGGAGTCGGAGAT	GCCGAGGACTTGAACTTCACTCG
Gapdh	GAPDH	AGGTCGGTGTGAACGGATTTG	TGTAGACCATGTAGTTGAGGTCA

Supplemental Table 2. The details of statistics used in this study.

Figure	Panel	Number of sample	Test used	F/t/p value and degrees of freedom (df)	Post hoc test	Significance
1A	cFos mRNA	Binge 0min = 6, Control 0min= 5<br>Binge 30min = 6, Control 30min= 4<br>Binge 120min = 5, Control120min= 5	Two-way factorial ANOVA	Time: F(2, 25) = 30.185, p < 0.05<br>Group: F(1, 25) = 7.945, p < 0.05<br>Interaction: F(2, 25) = 4.414, p < 0.05<br><br>Binge: F(2, 25) = 33.408, p < 0.05<br>Control: F(2, 25) = 5.401, p < 0.05	Sidak's multiple comparison	Binge vs Control (30 min), p < 0.05<br>0 min vs 30 min (Binge), p < 0.05<br>0 min vs 120 min (Binge), p < 0.05<br>0 min vs 30 min (Control), p < 0.05
1B	ΔFosB mRNA	Binge 0min = 8, Control 0min= 5<br>Binge 30min = 6, Control 30min= 4<br>Binge 120min = 5, Control120min= 4	Two-way factorial ANOVA	Time: F(2, 26) =11.843, p < 0.05<br>Group: F(1, 26) = 5.188, p < 0.05<br>Interaction: F(2, 26) = 1.638, ns<br><br>Binge: F(2, 26) =13.137, p < 0.05	Sidak's multiple comparison	0 min vs 120 min (Binge), p < 0.05
1C	pDARPP32/<br>tDARPP32	0 min = 7, 30 min = 5	Unpaired t-test	t = -2.994, df = 10		0 min vs 30 min, p < 0.05
1D	pDARPP32/<br>tDARPP32	0 min = 6, 30 min = 6	Unpaired t-test	t = -0.160, df = 10		0 min vs 30 min, p = 0.876, ns
1E	cFos mRNA	Binge 0min = 6, Control 0min= 5<br>Binge 30min = 7, Control 30min= 5<br>Binge 120min = 5, Control120min= 4	Two-way factorial ANOVA	Time: F(2, 26) = 34.415, p < 0.05<br>Group: F(1, 26) = 27.569, p < 0.05<br>Interaction: F(2, 26) = 10.682, p < 0.05<br><br>Binge: F(2, 26) = 47.562, p < 0.05	Sidak's multiple comparison	Binge vs Control (30 min), p < 0.05<br>0 min vs 30 min (Binge), p < 0.05<br>0 min vs 120 min (Binge), p < 0.05
1F	ΔFosB mRNA	Binge 0min = 5, Control 0min= 5<br>Binge 30min = 7, Control 30min= 4<br>Binge 120min = 5, Control120min= 4	Two-way factorial ANOVA	Time: F(2, 24) = 6.283, p < 0.05<br>Group: F(1, 24) =23.067, p < 0.05<br>Interaction: F(2, 24) = 3.084, ns<br><br>Binge: F(2, 24) = 9.956, p < 0.05	Sidak's multiple comparison	0 min vs 30 min (Binge), p < 0.05<br>0 min vs 120 min (Binge), p < 0.05
1G	pDARPP32/<br>tDARPP32	0 min = 7, 30 min = 7	Unpaired t-test	t = -2.344, df = 12		0 min vs 30 min, p < 0.05
1H	pDARPP32/<br>tDARPP32	0 min = 6, 30 min = 5	Unpaired t-test	t =1.058 , df = 9		0 min vs 30 min, p = 0.318, ns
2A	cFos mRNA	WT 0 min = 8, KO 0 min = 7,<br>WT 30 min =8, KO 30 min = 7	Two-way factorial ANOVA	Time: F(1, 26) = 108.197, p < 0.05<br>Group: F(1, 26) = 8.510, p < 0.05<br>Interaction: F(1, 26) = 5.579, p < 0.05<br><br>WT: F(1, 26) = 34.627, p < 0.05<br>KO: F(1, 26) = 76.367, p < 0.05	Sidak's multiple comparison	0 min vs 30 min (WT), p < 0.05<br>0 min vs 30 min (KO), p <0.05<br>WT vs KO (30min), p < 0.05
2B	ΔFosB mRNA	WT 0 min = 7, KO 0 min = 6,<br>WT 30 min =7, KO 30 min = 6	Two-way factorial ANOVA	Time: F(1, 22) = 14.982, p < 0.05<br>Group: F(1, 22) = 16.762, p < 0.05<br>Interaction: F(1, 22) = 4.828, p < 0.05<br><br>KO: F(1, 22) =17.095, p < 0.05	Sidak's multiple comparison	0 min vs 30 min (KO), p<0.05<br>WT vs KO (30min), p < 0.05

2C	pDARPP32/<br>tDARPP32 30 min	WT = 6, KO = 8	Unpaired t-test	t = -2.610, df = 7.651		WT vs KO, p < 0.05
2D	cFos mRNA	WT 0 min = 5, KO 0 min = 5,<br>WT 30 min =7, KO 30 min = 7	Two-way factorial ANOVA	Time: F(1, 20) = 73.608, p < 0.05<br>Group: F(1, 20) = 0.201, ns<br>Interaction: F(1, 20) = 0.666, ns<br><br>WT: F(1, 20) = 44.140, p < 0.05<br>KO: F(1, 20) = 30.134, p < 0.05	Sidak's multiple comparison	0 min vs 30 min (WT), p < 0.05<br>0 min vs 30 min (KO), p < 0.05
2E	ΔFosB mRNA	WT 0 min = 5, KO 0 min = 5,<br>WT 30 min =7, KO 30 min = 7	Two-way factorial ANOVA	Time: F(1, 20) = 13.620, p < 0.05<br>Group: F(1, 20) = 0.20, ns<br>Interaction: F(1, 20) =0.451, ns<br><br>WT: F(1, 20) = 9.515, p < 0.05<br>KO: F(1, 20) = 4.556, p < 0.05	Sidak's multiple comparison	0 min vs 30 min (WT), p < 0.05<br>0 min vs 30 min (KO), p < 0.05
2F	pDARPP32/<br>tDARPP32 30 min	WT = 6, KO = 8	Unpaired t-test	t = -1.3327, df = 12		WT vs KO, p =0.209, ns
3B	Cell counts	WT 0 min = 4, KO 0 min = 4,<br>WT 30 min = 7, KO 30 min = 7	Two-way factorial ANOVA	Genotype: F(1, 18) = 21.597,p < 0.05<br>Time: F(1, 18) = 449.647, p < 0.05<br>Interaction: F(1, 18) = 24.767, p < 0.05	Sidak's multiple comparison	WT 0 min vs WT 30 min, p < 0.05<br>KO 0 min vs KO 30 min, p < 0.05<br>WT 30 min vs KO 30 min, p < 0.05
3D	Cell counts	WT 0 min = 4, KO 0 min = 4,<br>WT 30 min = 7, KO 30 min = 8	Two-way factorial ANOVA	Genotype: F(1, 19) = 0.116, p =0.738, ns<br>Time: F(1, 19) = 29.635,p < 0.05<br>Interaction: F(1, 19) = 0.493, p =0.491, ns	Sidak's multiple comparison	WT 0 min vs WT 30 min, p < 0.05<br>KO 0 min vs KO 30 min, p < 0.05
4A	cFos mRNA	Vehicle 0min = 10, Baclofen 0min= 4<br>Vehicle 30min = 10, Baclofen 30min= 9<br>Vehicle 120min = 10, Baclofen 120min= 6	Two-way factorial ANOVA	Time: F(2, 43) = 15.781, p < 0.05<br>Group: F(1, 43) = 13.305, p < 0.05<br>Interaction: F(2, 43) = 3.790, p < 0.05<br><br>Vehicle: F(2, 43) = 24.573, p < 0.05<br>Baclofen: F(2, 43) = 1.740, ns	Sidak's multiple comparison	Vehicle vs Baclofen (30 min), p < 0.05<br>Vehicle vs Baclofen (120 min), p < 0.05<br>0 min vs 30 min (Vehicle), p < 0.05<br>0 min vs 120 min (Vehicle), p < 0.05
4B	ΔFosB mRNA	Vehicle 0min = 12, Baclofen 0min= 4<br>Vehicle 30min = 10, Baclofen 30min= 11<br>Vehicle 120min = 10, Baclofen 120min= 6	Two-way factorial ANOVA	Time: F(2, 47) = 4.766, p < 0.05<br>Group: F(1, 47) = 8.655, p < 0.05<br>Interaction: F(2, 47) =1.320, ns<br><br>Vehicle: F(2, 47) = 8.310, p < 0.05<br>Baclofen: F(2, 47) = 0.977, ns	Sidak's multiple comparison	Vehicle vs Baclofen (30 min), p < 0.05<br>Vehicle vs Baclofen (120 min), p < 0.05<br>0 min vs 120 min (Vehicle), p < 0.05
4C	pDARPP32/<br>tDARPP32 30 min	Vehicle = 9, Baclofen = 10	Unpaired t-test	t = 2.501, df = 17		Vehicle vs Baclofen, p < 0.05
4D	cFos mRNA	Vehicle 0min = 6, Baclofen 0min= 5<br>Vehicle 30min = 6, Baclofen 30min= 6<br>Vehicle 120min = 5, Baclofen 120min= 6	Two-way factorial ANOVA	Time: F(2, 28) = 62.706, p < 0.05<br>Group: F(1, 28) = 1.434, ns<br>Interaction: F(2, 18) = 1.780, ns<br><br>Vehicle :F(2, 28) =40.215, p <0.05<br>Baclofen: F(2, 28) =25.059, p <0.05	Sidak's multiple comparison	0 min vs 30 min (Vehicle), p < 0.05<br>0 min vs 120 min (Vehicle), p < 0.05<br>0 min vs 30 min (Baclofen), p < 0.05<br>0 min vs 120 min (Baclofen), p < 0.05

4E	ΔFosB mRNA	Vehicle 0min = 5, Baclofen 0min= 5<br>Vehicle 30min = 6, Baclofen 30min= 6<br>Vehicle 120min = 5, Baclofen 120min= 6	Two-way factorial ANOVA	Time: F(2, 27) = 12.463, p < 0.05<br>Group: F(1, 27) = 0.64, ns<br>Interaction: F(2, 27) = 0.153, ns<br><br>Vehicle :F(2, 27) =5.396, p <0.05<br>Baclofen: F(2, 27) =7.314, p <0.05	Sidak's multiple comparison	0 min vs 30 min (Vehicle), p < 0.05<br>0 min vs 120 min (Vehicle), p < 0.05<br>0 min vs 30 min (Baclofen), p < 0.05<br>0 min vs 120 min (Baclofen), p < 0.05
4F	pDARPP32/<br>tDARPP32 30 min	Vehicle = 7, Baclofen = 7	Unpaired t-test	t = 0.988, df = 12		Vehicle vs Baclofen, p < 0.342, ns
5A	cFos mRNA 30 min	WT-vehicle = 16, WT-baclofen = 15,<br>KO-vehicle = 15, KO-baclofen = 10	Two-way factorial ANOVA	Genotype: F(1, 52) = 14.461, p < 0.05<br>Treatment: F(1, 52) = 6.105, p < 0.05<br>Interaction: F(1, 52) = 5.218, p < 0.05	Sidak's multiple comparison	WT-vehicle vs WT-baclofen, p < 0.05<br>WT-baclofen vs KO-baclofen, p < 0.05
5B	ΔFosB mRNA 30 min	WT-vehicle = 14, WT-baclofen = 17,<br>KO-vehicle = 15, KO-baclofen = 10	Two-way factorial ANOVA	Genotype: F(1, 52) = 32.015, p < 0.05<br>Treatment: F(1, 52) = 4.969, p < 0.05<br>Interaction: F(1, 52) = 4.373, p < 0.05	Sidak's multiple comparison	WT-vehicle vs WT-baclofen, p < 0.05<br>WT-vehicle vs KO-vehicle, p < 0.05<br>WT-baclofen vs KO-baclofen, p < 0.05
5C	pDARPP32/<br>tDARPP32 30 min	WT-vehicle = 6, WT-baclofen = 9,<br>KO-vehicle = 6, KO-baclofen = 6	Two-way factorial ANOVA	Genotype: F(1, 23) = 12.487, p < 0.05<br>Treatment: F(1, 23) = 4.373, p < 0.05<br>Interaction: F(1, 23) = 5.514, p < 0.05	Sidak's multiple comparison	WT-vehicle vs WT-baclofen, p < 0.05<br>WT-baclofen vs KO-baclofen, p < 0.05
5D	cFos mRNA 30 min	WT-vehicle = 6, WT-baclofen = 6,<br>KO-vehicle = 7, KO-baclofen = 5	Two-way factorial ANOVA	Genotype: F(1, 20) = 0.157, ns<br>Treatment: F(1, 20) = 3.815, ns<br>Interaction: F(1, 20) = 0.434, ns		
5E	ΔFosB mRNA 30 min	WT-vehicle = 6, WT-baclofen = 6,<br>KO-vehicle = 7, KO-baclofen = 5	Two-way factorial ANOVA	Genotype: F(1, 20) = 0.419, ns<br>Treatment: F(1, 20) = 1.959, ns<br>Interaction: F(1, 20) = 0.078, ns		
5F	pDARPP32/<br>tDARPP32 30 min	WT-vehicle = 8, WT-baclofen = 8,<br>KO-vehicle = 6, KO-baclofen = 6	Two-way factorial ANOVA	Genotype: F(1, 24) = 0.260, ns<br>Treatment: F(1, 24) = 0.006, ns<br>Interaction: F(1, 24) = 0.531, ns		
6B	Cell counts	WT-vehicle = 7, WT-baclofen = 4,<br>KO-vehicle = 7, KO-baclofen = 4	Two-way factorial ANOVA	Genotype: F(1, 18) = 57.057, p < 0.05<br>Treatment: F(1, 18) = 1.581, NS<br>Interaction: F(1, 18) = 8.184, p < 0.05	Sidak's multiple comparison	WT-vehicle vs WT-baclofen, p < 0.05<br>WT-vehicle vs KO-vehicle, p < 0.05<br>WT-baclofen vs KO-baclofen, p < 0.05
6D	Cell counts	WT-vehicle = 7, WT-baclofen = 7,<br>KO-vehicle = 6, KO-baclofen = 5	Two-way factorial ANOVA	Genotype: F(1, 21) = 3.017, ns<br>Treatment: F(1, 21) = 0.005, ns<br>Interaction: F(1, 21) = 0.402,ns		
S1B	Body weight	Binge = 5,Control = 6	Unpaired t-test	t = 0.581, df = 9		Control vs Binge, p < 0.576, ns
S1C	Food intake	Binge = 8,Control = 6	Two-way ANOVA assessed<br>by repeated measures	Time: F(2, 24) = 724.546, p < 0.05<br>Group: F(1, 12) = 233.007, p < 0.05<br>Interaction: F(2, 24) = 116.692, p < 0.05	Sidak's multiple comparison	Binge vs Control (30 min), p < 0.05<br>Binge vs Control (120 min), p < 0.05
S1D	HFD intake	WT=10	Pearson's correlation coefficient	r=0.673, p<0.05		
S1E	cFos mRNA	Binge 0min = 9, Control 0min= 9<br>Binge 30min = 6, Control 30min= 5<br>Binge 120min = 4, Control120min= 5	Two-way factorial ANOVA	Time: F(2, 32) = 48.977, p < 0.05<br>Group: F(1, 32) = 2.262, ns<br>Interaction: F(2, 32) = 0.969, ns<br><br>Binge: F(2, 32) = 21.725, p < 0.05<br>Control: F(2, 32) = 28.739, p < 0.05	Sidak's multiple comparison	0 min vs 30 min (Binge), p < 0.05<br>0 min vs 30 min (Control), p < 0.05

S1F	ΔFosB mRNA	Binge 0min = 4, Control 0min= 4<br>Binge 30min = 6, Control 30min= 5<br>Binge 120min = 4, Control120min= 5	Two-way factorial ANOVA	Time: F(2, 22) = 24.635, p < 0.05<br>Group: F(1, 22) =3.250, ns<br>Interaction: F(2, 22) = 0.596, ns<br><br>Binge: F(2, 22) = 13.274, p < 0.05<br>Control: F(2, 22) = 11.967, p < 0.05	Sidak's multiple comparison	0 min vs 120 min (Binge), p < 0.05<br>0 min vs 120 min (Control), p < 0.05
S1G	DRD1/β actine	0 min = 6, 30 min = 6	Unpaired t-test	t = 0.802, df = 5.976		0 min vs 30 min, p = 0.453, ns
S1H	DRD1/β actine	0 min = 6, 30 min = 6	Unpaired t-test	t = 1.454, df = 10		0 min vs 30 min, p = 0.177, ns
S2A	Food intake	WT = 11, KO = 5	Two-way ANOVA assessed by repeated measures	Time: F(2, 28) = 638.689, p < 0.05<br>Group: F(1, 14) = 15.099, p < 0.05<br>Interaction: F(2, 28) = 6.042, p < 0.05	Sidak's multiple comparison	WT vs KO (30 min), p < 0.05<br>WT vs KO (120 min), p < 0.05
S2B	DRD1/β actine	WT = 6, KO = 6	Unpaired t-test	t = 0.630, df = 10		0 min vs 30 min, p = 0.543, ns
S2C	DRD1/β actine	WT = 6, KO = 6	Unpaired t-test	t = 0.225, df = 10		0 min vs 30 min, p = 0.827, ns
S3A	Cell counts	WT 0 min = 4, KO 0 min = 4,<br>WT 30 min = 6, KO 30 min = 7	Two-way factorial ANOVA	Genotype: F(1, 17) = 0.317, ns<br>Time: F(1, 17) = 38.453, p < 0.05<br>Interaction: F(1, 17) = 0.317, ns	Sidak's multiple comparison	WT 0 min vs WT 30 min, p < 0.05<br>KO 0 min vs KO 30 min, p < 0.05
S3B	Cell counts	WT 0 min = 4, KO 0 min = 4,<br>WT 30 min = 7, KO 30 min = 8	Two-way factorial ANOVA	Genotype: F(1, 19) = 0.017,ns<br>Time: F(1, 19) = 24.498, p < 0.05<br>Interaction: F(1, 19) = 0.927, ns	Sidak's multiple comparison	WT 0 min vs WT 30 min, p < 0.05<br>KO 0 min vs KO 30 min, p < 0.05
S4A	Food intake	WT-vehicle = 9, WT-baclofen = 10,<br>KO-vehicle = 9, KO-baclofen = 7	Two-way ANOVA assessed by repeated measures	Time: F(2, 62) = 910.077, p < 0.05<br>Treatment: F(3, 31) = 16.012, p < 0.05<br>Interaction: F(6, 62) = 10.108, p < 0.05	Sidak's multiple comparison	WT-vehicle vs WT-baclofen (30 min), p < 0.05<br>WT-vehicle vs KO-vehicle (30 min), p < 0.05<br>WT-vehicle vs WT-baclofen (120 min), p < 0.05
S5A	Cell counts	WT-vehicle = 7, WT-baclofen = 4,<br>KO-vehicle = 6, KO-baclofen = 4	Two-way factorial ANOVA	Genotype: F(1, 17) = 0.02, p ns<br>Treatment: F(1, 17) = 0.178, ns<br>Interaction: F(1, 17) = 0.404, ns		
S5B	Cell counts	WT-vehicle = 7, WT-baclofen = 7,<br>KO-vehicle = 6, KO-baclofen = 5	Two-way factorial ANOVA	Genotype: F(1, 21) = 1.487, ns<br>Treatment: F(1, 21) = 0.659, ns<br>Interaction: F(1, 21) = 0.015, ns		
